# The ipsilateral silent period: an early diagnostic marker of callosal disconnection in ALS

**DOI:** 10.1177/20406223211044072

**Published:** 2021-09-13

**Authors:** Annemarie Hübers, Jan Kassubek, Hans-Peter Müller, Nicolas Broc, Jens Dreyhaupt, Albert C. Ludolph

**Affiliations:** Department of Clinical Neurosciences, Geneva University Hospitals, Rue Gabrielle-Perret-Gentil 4, 1205 Geneva, Switzerland; Department of Neurology, University Hospital Ulm, Ulm, Germany; Department of Neurology, University Hospital Ulm, Ulm, Germany; Department of Clinical Neurosciences, Geneva University Hospitals, Geneva, Switzerland; Institute of Epidemiology and Medical Biometry, University of Ulm, Ulm, Germany; Department of Neurology, University Hospital Ulm, Ulm, Germany

**Keywords:** amyotrophic lateral sclerosis, corpus callosum, mirror movements, transcranial magnetic stimulation

## Abstract

**Introduction:**

Imaging studies showed affection of the corpus callosum (CC) in amyotrophic lateral sclerosis (ALS). Here, we sought to determine whether these structural alterations reflect on the functional level, using transcranial magnetic stimulation (TMS).

**Methods:**

In 31 ALS patients and 12 controls, we studied mirror movements (MM) and transcallosal inhibition (TI) using TMS. Structural integrity of transcallosal fibres was assessed using diffusion tensor imaging.

**Results:**

TI was pathologic in 25 patients (81%), 22 (71%) showed MM. Loss of TI was observed in very early stages (disease duration <4 months). No correlation was found between TI/MM and fractional anisotropy of transcallosal fibres.

**Discussion:**

These results substantiate the body of evidence towards a functional involvement of the CC in early ALS beyond microstructural alterations.

**Significance:**

TI may become a useful early diagnostic marker in ALS, even before descending tracts are affected. Diagnostic delay in ALS is high, often preventing patients from gaining access to therapeutic trials, and sensitive diagnostic tools are urgently needed. Our findings also provide insights into the pathophysiology of ALS, potentially supporting the so-called ‘top-down’ hypothesis, that is, corticoefferent (intracortical/corticospinal) propagation. Callosal affection in early stages might represent the ‘missing link’ to explain corticocortical disease-spreading.

## Introduction

Amyotrophic lateral sclerosis (ALS) is a fatal adult onset motor neuron disease, simultaneously affecting upper motor neurons and lower motor neurons in the brain and spinal cord [central nervous system (CNS) and peripheral nervous system (PNS)].^1,2^ Yet, not only the motor neurons are affected: during the last two decades, the notion of ALS as a multisystem disorder has become more and more prominent.^3^ Neuropathological studies showed the accumulation of misfolded protein aggregates in multiple cortical and subcortical areas of the CNS.^4^ Imaging studies could demonstrate that the motor part of the corpus callosum (CC), being one of the main fibre tracts within the CNS, is also affected in ALS.^5–8^ Yet, it remains unclear how these alterations are reflected on the functional level.

Transcallosal inhibition (TI) represents the functional integrity of callosal motor fibres and can be noninvasively studied by means of transcranial magnetic stimulation (TMS). While double-pulse protocols using different stimulus intensities can be rather time-consuming and sometimes a strain for the patient, the ipsilateral silent period (iSP) can be studied using a short single-pulse TMS protocol. ISP is thought to be mediated *via* transcallosal inhibitory neurons from the stimulated (i.e. active) to the nonstimulated primary motor cortex^9,10^ and is a marker of the functional integrity of callosal motor fibres. Reduction of functional TI has been reported in ALS patients, but the electrophysiological findings seem not to be associated with a decrease of fractional anisotropy (FA) in the motor region of the CC.^11,12^

With this study, we intended to substantiate the body of evidence pointing towards a functional involvement of transcallosal motor fibres in ALS, as described in a previous study from our group,^6^ using a single-coil TMS protocol that can easily be implied in the clinical diagnostic workup, which we combined with diffusion tensor imaging (DTI)-based microstructural measures of the CC.

## Materials and methods

We included 31 ALS patients [11 women, mean age: 63 ± 12 years, median disease duration: 12.0 months (interquartile range [IQR]: 8–18 months), 8 bulbar onset] and 12 age-matched controls (five women, mean age: 60 ± 12 years). Out of these, 19 patients underwent DTI (four women, mean age: 65 ± 14 years). All participants gave their written informed consent. This is a nonblinded, nonrandomized, controlled, prospective, noninterventional study. The chosen sample number was considered as adequate to provide answers to the specific questions of this study, although a formal samples size calculation could not be performed before study start. Additional ‘non-statistical’ criteria, namely the incidence of the studied disease, had to be taken into consideration. The study was approved by the local Ethics Committee of the University of Ulm, Germany (reference number: 210/17). All patients were diagnosed according to the revised El Escorial criteria.^13^ The controls had no history of neurological or psychiatric disease. All patients and controls were right handed with a lateralization index of greater than 0.4 according to the Edinburgh Handedness Inventory.^14^ Additional inclusion criteria were as follows: age over 18 years, capability of thoroughly understanding all information given and giving full informed consent.

The clinical disease burden was objectified using the revised ALS functional rating scale (ALS-FRS-R).^15^

Mirror movements (MM) were studied clinically in both hands according to an established protocol.^16^ Patients were instructed to perform ballistic extensions of the fingers of one hand (task hand) at a self-paced rate of approximately 0.5 Hz while focussing visually on this hand and relaxing the contralateral hand (mirror hand). Involuntary visible coactivation (i.e. extension of the fingers by activating the small intrinsic hand muscles, with a smaller amplitude of movement compared with the voluntary active side) of the mirror hand while performing this task was recorded by the investigator and, if present, classified as overt MM.

ISP was studied in the abductor pollicis brevis (APB) muscle of each hand using a figure-of-eight shaped coil (outer diameter of each wing, 90 mm) that was connected to a Magstim 200 stimulator (The Magstim Company, Whitland, UK). The surface electromyogram (EMG) was recorded using disposable electrodes (Silver Mactrode Plus, Leonhard Lang GmbH, Innsbruck, Austria) in a belly-tendon montage. The EMG was band-pass filtered and digitized at a sampling rate of 50,000 Hz (Neurowerk EMG, SIGMA Medizintechnik, Gelenau, Germany). The TMS stimulus was applied during maximal voluntary contraction of the APB. The online display of the EMG signal served as feedback for the investigator and the loudspeaker as feedback for the patients. The coil was placed tangentially to the scalp. The optimal stimulation site was defined as the site that produced consistently largest motor evoked potential (MEP) in the relaxed APB of the contralateral hand. We chose the APB as target muscle because in other small hand muscles, in particular in the first dorsal interosseous (FDI) muscle, a second phase of inhibition that is most likely mediated *via* the ipsilateral corticospinal tract (CST) may obscure the iSP. This second phase of inhibition is absent in the APB.^10,17^ The intensity of the TMS stimulus was set at 140% of the resting motor threshold (RMT).^17^ RMT was defined as the minimum stimulus intensity that elicited MEPs greater than 50 µV in at least 5 out of 10 consecutive trials.^18^ Ten trials were recorded, rectified and superimposed for either cortex. Short pauses between the trials were allowed to prevent muscle fatigue. ISP onset latency and iSP duration were measured for either hand and individual using a graphical method.^19^ As normal values, an upper limit of 36.2 ms for iSP onset latency and 10–40 ms for its duration were set, as previously published in healthy elderly patients.^20–22^

The central motor conduction time (CMCT) to the APB of both hands was studied in ALS patients using the F-wave method. CMCT was calculated as the difference between the corticomuscular latency (CML) and the peripheral muscular latency (PML): CMCT = CML − PML; PML was calculated as follows: (F-wave latency + M-wave latency − 1) / 2, with 1 being the estimated delay at the α motoneuron for antidrome stimulation.

DTI scanning was performed by multislice single-shot two-dimensional (2D) spin-echo echo planar imaging (EPI) on a 3.0 Tesla Achieva whole-body system (Philips Medical System, Best, The Netherlands); the DTI protocol consisted of 16 volumes (60 slices, 112 × 112 pixels, slice thickness: 2.0 mm, pixel size: 2.0 mm × 2.0 mm) representing 15 gradient directions (*b* = 1000 s/mm^2^) and one scan with gradient 0 (*b* = 0). The echo time (TE) and repetition time (TR) were 70 and 9881 ms, respectively. Fluid-attenuated inversion recovery (FLAIR) imaging was acquired in addition to control for the presence of parenchymal (especially microvascular) brain lesions.

The DTI analysis was performed by use of analysis software tensor imaging and fibre tracking (TIFT).^23^ After spatial normalization to the Montreal Neurological Institute (MNI) stereotaxic space, calculated FA maps were smoothed with a Gaussian filter of 8-mm full width at half maximum (FWHM).^24^

### Statistical analysis

To identify a typical ALS-associated alteration pattern, the 19 DTI data sets of ALS patients were compared with an age- and sex-matched sample of 19 DTI data sets of controls from our DTI database. Statistical comparison by Student’s *t* test was performed voxelwise for FA values to detect changes between the subject groups [whole brain-based spatial statistics, (WBSS)].^24^ Voxels with FA values below 0.2 were not considered for statistical comparison because cortical grey matter shows FA values up to 0.2. Statistical results were corrected for multiple comparisons using the false discovery rate (FDR) algorithm at *p* < 0.05.^25^ Further reduction of the alpha error was performed by a spatial correlation algorithm that eliminated isolated voxels or small isolated groups of voxels in the size range of the smoothing kernel leading to a threshold cluster size of 256 voxels.

Voxelwise association analysis of FA values to iSP measures was performed by Pearson correlation. Statistical results were then corrected for multiple comparisons using the FDR algorithm at *p* < 0.05,^25^ as well as by spatial voxel clustering.^23,24^

Statistical analysis was performed by SAS version 9.4 under Windows. Continuous variables were described as mean ± standard deviation or median together with IQR as appropriate. Categorical variables were described as absolute and relative frequencies, respectively. Fisher’s exact test was used to evaluate differences of iSP measures and MM between ALS patients and controls. The two-sample *t* test or Wilcoxon rank sum test as appropriate was used to investigate group differences in continuous variables. Associations between iSP and FA of the motor part of the CC were investigated using scatter plots and the Spearman’s rank correlation coefficient. A two-sided *p* value of less than 0.05 was considered statistically significant. An adjustment for multiple testing was not done. Due to the explorative nature of this study, all results from statistical tests have to be interpreted as hypothesis generating.

## Results

Mean CMCT of ALS patients was 7.4 ± 1.8 ms to the left APB and 7.3 ± 2.0 ms to the right APB. Mean RMT did not differ significantly between patients and controls: 63.0 ± 17.3 in patients *versus* 60.0 ± 14.0 in controls for the right hemisphere and 61.4 ± 20.9 in patients *versus* 58.4 ± 12.9 in controls for the left hemisphere. That way, differences in relative stimulus intensity (i.e. 140% of individual RMT) could be ruled out as reason for differences in iSP between both groups.

**Figure 1.**
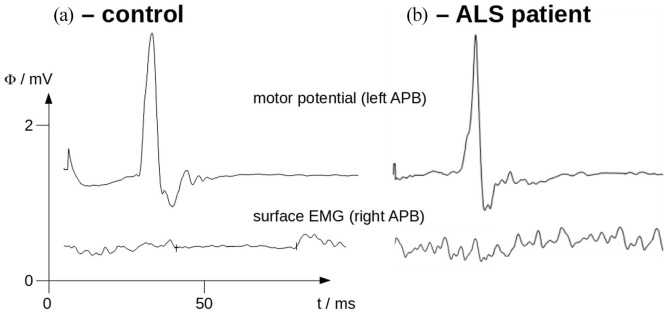
Motor potentials evoked in the left APB (upper row) and surface EMG of the right APB (lower row) during maximal voluntary contraction (averaged EMG of 10 trials) of a control (a) and an ALS patient (b). Note the complete loss of iSP (lower row) in the ALS patient, while present and within normal ranges regarding onset latency, depth and duration in the control. ALS, amyotrophic lateral sclerosis; APB, abductor pollicis brevis; EMG, electromyogram; iSP, ipsilateral silent period.

ISP was pathologic in 25 out of 31 ALS patients (81%), that is, prolongation or complete loss in one or both hemispheres. Figure 1 shows two representative examples of electrophysiological measurements in a patient and a control.

We found a significant difference of iSP in patients *versus* controls if the ‘inhibiting’ cortex was the dominant (left) one (*p* = 0.01). For iSP ipsilateral to the right hemisphere, we observed a nonsignificant trend towards decreased inhibition in ALS patients (*p* = 0.06). In addition, we observed significantly more MM, which were present in 22 (71%) of ALS cases (*p* < 0.01). ISP loss was present in early (i.e. 3–7 months since symptom onset) as well as late stages of the disease.

No correlation was observed between site of onset (bulbar *versus* spinal) and iSP loss, nor with ALS-FRS-R.^15^

**Figure 2.**
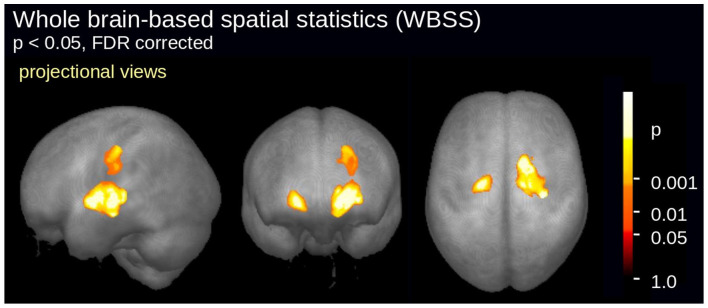
WBSS of fractional anisotropy maps of 19 ALS patients *versus* 19 controls. Significant alterations were detected along the corticospinal tracts. ALS, amyotrophic lateral sclerosis; FDR, false discovery rate.

WBSS of FA maps of 19 ALS patients *versus* 19 controls revealed a significant alteration pattern bihemispherically along the CSTs (Figure 2). This pattern has been shown to be characteristic for ALS at the group level.^26,27^ Although the group of ALS patients studied here demonstrated this typical ALS-associated alteration pattern in the whole brain-based DTI analysis, no significant correlation could be observed between electrophysiological measurements of the motor CC (iSP) and FA of its motor part in ALS patients.

## Discussion

In this study, we provide evidence that the CC is functionally affected in ALS patients in early as well as late stages of the disease. ALS patients exhibited reduced transcallosally mediated functional communication between both primary motor cortices, reflecting as overt MM on the clinical level.

No correlation between callosal function and diffusion imaging measures could be observed. These results are in line with previous studies^6,11,12^ and suggest that functional disturbance might precede detectable microstructural abnormalities. Of note, patients in our cohort showed no alterations of the CST (i.e. normal CMCT)^28^ nor of the motor threshold. Thus, loss of callosal inhibition may be regarded as a very sensitive and early marker of disease activity, unmasking cortical changes in ALS before they can be detected by other electrophysiological or imaging methods. The functional affection of the CC in early stages of the disease might also be the ‘missing link’ to explain the spreading of TAR DNA-binding protein 43 (TDP-43) pathology from one hemisphere to another, which could not yet be fully explained by neuropathological studies.

We suggest that this protocol, which does not require complete relaxation of the target muscle and takes only about 15 min for an experienced investigator to perform, can easily be implemented in the clinical routine diagnostic workup in ALS.

In addition, iSP study may be of potential use for diagnosis and better characterization of ALS patients to be recruited in clinical trials. In case of delayed diagnosis, institution of appropriate management strategies, such as commencement of neuroprotective therapies, may be critically delayed, and recruitment into clinical trials may occur at later stages in the disease process, perhaps beyond the therapeutic window period. TMS parameters may be applied as biomarkers in therapeutic ALS trials. In fact, assessing the biological effects of future neuroprotective agents on TMS outcome parameters could potentially determine therapeutic efficacy at an early stage of drug development, thereby preventing unnecessary and costly phase III trials.

The main limitation of our study is the relatively small sample size and the fact that no longitudinal examinations were performed. In addition, we did not compare ALS patients with patients with other motor neurone disease (MND) or frontal dementia. Studies in larger patient cohorts will be necessary to further address these issues.

## Conclusion

The CC is functionally affected in ALS patients in early disease stages. Our results provide important insight into the pathophysiological mechanisms underlying degeneration of the CNS in MND. Investigation of CC function by means of TMS may be a useful tool to reduce diagnostic delay. In addition, it may also be used as a potential biomarker in clinical therapeutic trials.
